# The Immediate Removal of the Secundines after Abortion

**Published:** 1884-02-20

**Authors:** Arch Dixon

**Affiliations:** Henderson, Ky.


THE
### Southern Medical Record:

EDITORS:
THOMAS S. POWELL, M.D. R. C. WORD, M.D.

*R. C. WORD, M.D., Managing Editor.*

☞All Communications and Letters on Business connected with the RECORD must be addressed to the Managing Editor.

VOL. XIV. ATLANTA, GA., FEBRUARY 20, 1884. No. 2.

### ORIGINAL AND SELECTED ARTICLES.

### THE IMMEDIATE REMOVAL OF THE SECUNDINES AFTER ABORTION.

BY ARCH DIXON, M.D., HENDERSON, KY.

The main indications for the treatment of abortion are clearly enough set forth in our systematic Treatises on Midwifery in present use in our schools, and several points have recently been prominently discussed in obstetrical and gynecological societies and medical journals, relating to a better control of hemorrhage, and the necessity of greater care in the after treatment of women who have recently miscarried, as a means of prophylaxis against the occurrence of diseases requiring, later on, the services of the gynecologist.

Playfair lays some stress on this subject, and declares that sufficient attention is not devoted to this very important point of treatment, and thinks it a frequent source of trouble on the part of patients who have miscarried.

To one acquainted with the more recent obstetric literature, the treatment of abortion is not especially a vexed question until he arrives at that stage when the fœtus is expelled and the secundines are retained. In cases of too early rupture of the amnion, as the result of strong uterine contractions, or too persistent digital manipulation, and a fœtus, say under the fourth month, is expelled; and the membranes are retained by the premature contraction of the internal os uteri, where little or no hemorrhage occurs; the patient is feeling comfortable and no immediate danger seems impending; the next question which needs authoritative solution is, what are we to do? What is the measure of our responsibility?

The patient and her family, reposing implicit confidence in the physician of their choice, rely confidently upon his advice and counsel, at such a time as this. He is supposed to know the dangers, and is looked to for such treatment and suggestions as will effectually ward off their approach, and restore the patient to her previous health, unimpaired by their menacing presence.

We are expected to look into the future and see the liability of the occurrence of hemorrhage, placental polyps, sub-involution, uterine displacements, and their attendant evils. They cannot foresee the dangers which may arise, and consequently trust to us to shield and protect them; and when it is advised that nothing further be done, that nature be trusted to expel in her own time and way, or perhaps to absorb, the contents of the uterus, they are well satisfied, and too often pleased to be let alone, resting in the security of our counsel.

Patients thus managed pass too frequently from the care of their medical attendants as cured, only to return months thereafter with one of the above-mentioned ailments for treatment, or into the hands of another physician who may or may not recognize the cause of the trouble.

Should he in his diagnostic investigations come upon portions of retained placenta or partially putrid membranes, the chances are that the patient will lose confidence in the physician through whose neglect, ignorance or inattention she has suffered so much pain, sickness and expense. Instead of the picture presenting itself in this light, hemorrhage, fetid discharges from the putrid secundines, chills, fevers, and septicæmia may early occur, and we may scarcely be able to save the life which we should never have allowed to be so seriously jeopardized.

I desire to discuss this question from the affirmative side, and to state as a general principle of sound treatment, that a woman is never safe until all matters connected with the pregnancy are expelled from the uterus, and that our chief indication for treatment is to cause their immediate removal, either manually or instrumentally.

Perhaps some apology may be due for bringing forward so stale a subject for your consideration, but from my recent reading, I feel compelled to state my belief that a large number of practitioners throughout the country—some of them among us—do not hold these views, or practice them in the treatment of their patients.

This is proved by the recent discussion of this subject in two very forcibly written papers, both published in the February number of the American Journal of Obstetrics, (1883), by T. Johoson Alloway, M. D., of Montreal, and Dr. Paul F. Munde, of New York.

In both these papers the importance of removing *at once* a retained placenta after abortion, is advocated. Dr. Munde, in support of the views advanced by Dr. Alloway, expresses himself thus strongly: "It is not my intention to go over the well-worn ground of the treatment, expectant and active, of retention of the membranes and placenta after a miscarriage. I have frequently expressed my convictions on this, both orally and in print, during the past ten years. and have become accustomed to meeting with the same objections from the older members of the profession, the general practitioners; and the same indorsement from the younger, more bold, and perhaps, more progressive physicians. I do not expect to alter the views of the former gentlemen who, in a practice of many years, have always found the "let alone" policy answer well, both for themselves and their patients. The settled convictions of years cannot be changed by the teachings or experience of others. But I wish to add my testimony to that of Dr. Alloway, as given in his paper, in the present number, in favor of the forcible (that is manual or instrumental) removal of the secundines immediately after the expulsion of the fœtus, in every case where the cervical canal is sufficiently patulous to permit the introduction of the finger or of the large dull curetto or the placental forceps. Further, if there is hemorrhage, or an offensive vaginal discharge, or if the temperature rises, or there is a chill, and the secundines are still retained, no matter how soon or how late after the expulsion of the fœtus, they should be at once removed, and if necessary the cervix dilated to facilitate the operation. I have seen exhausting, almost fatal hemorrhage continue for three weeks after a miscarriage, which was at once arrested on the removal of the retained placenta; and I have witnessed almost fatal septic infection come on within forty hours after expulsion of the fœtus, and fatal septicæmia from putrescence of the retained placenta, in spite of a temporary improvement after removal of that body on the fourth day. And I have seen women confined to their beds by hemorrhage, or annoyed for weeks by sanguineous oozing, worried in mind and weakened in body thereby, all because the placenta had been left to "come away" by itself. That these occurrences are not pictures of fancy, is known to every practitioner of experience. And that the anxiety of a woman who knows that her womb still contains a body which ought to and *must*, sooner or later, come away, or be removed by force, should be respected, as well as the undoubted danger from septicæmia and hemorrhage, which she incurs so long as the uterus is not emptied, seems so obvious as hardly to require renewed assertion."

Until recently the general teaching of text books has been in favor of temporizing. As far as they gave any definite instruction, it was to the effect that, if difficulty was encountered in the delivery of the after-birth or membranes, more injury was likely to result from the manipulation necessary for their removal than would occur if it was left alone and nature trusted to take care of it; the physician must therefore place a tampon in the vagina and wait for twelve or twenty-four hours, giving ergot in the meantime, and if the placenta be not then expelled, to tampon again, and so on *ad infinitum*.

Leishman says: "If therefore, the whole ovum is not expelled entire, the effect of the uterine contractions will be to rupture the membranes and expel the embryo through the cervix which has been sufficiently dilated for the purpose. The action then ceases, the os closes, and the placenta is retained. This being the state of matters, we can do nothing but wait, the safest course is that of an expectant attitude, when, after the interval of hours or days, hemorrhage may become so profuse as to require the plug while we wait for the dilatation of the os."

Dr. Alloway, commenting upon this teaching, says: "Here it is clearly shown that the entire cervix has become sufficiently dilated to allow of the exit of the embryo, and therefore of the entrance of the finger. And instead of endeavoring to empty the uterus while the cervix is dilated or dilatable, we are quietly to sit down and wait until our unfortunate patient has *almost* bled to death, and the placenta, enclosed as a foreign body in the uterus, has undergone decomposition and given rise to septicæmia."

Very different from the ideas of Leishman are those of Angus McDonald, President of the Obstetical Society of Edinburgh, as expressed in a paper published in The Lancet, in February, 1880. He says: "I believe also that more harm is likely to result from under-caution than from undue interference in such cases; at least experience has led me to believe that a majority of our professional brethren are more apt to err in this particular, through defect than through excess of activity. The result is that their patients are liable to be landed in troubles that are much more serious than any dangers, real or imaginary, connected with a thorough evacuation of the uterus. To put it plainly, I believe a patient is put to a greaetr risk from a portion of the membranes being left in the uterus to become organized and a source of persistent menorrhagia than one is likely to suppose from the manipulation necessary to secure complete removal of every part of the ovum in miscarriage."

The clinical history of cases is better evidence than any amount of theory, and I therefore present, briefly, two or three cases coming under my own observation :

CASE 1. In October last, I was called to see Emily B—. She had been under the care of Mrs. Rhodes. I found that she had been delivered of a fœtus, at about the third month of pregnancy, ten days previous to my call. Nothing unusual occured during the labor. She lost no great quantity of blood, the fœtus had been expelled without difficulty, but the membranes were retained ; considerable ergot had been given,severe pains were produced but nothing was discharged from the uterus. She did well for several days. In about a week, however, the discharges became offensive. She had repeated chills, night sweats, fever, furred tongue, high temperature, anorexia, and exhausting diarrhœa, no pain. Upon my first visit, after learning these facts, I dilated the cervix and removed a most offensive mass from the uterus, washed out its cavity with carbolized water, and placed her upon a nourishing diet, quinine, opium, etc. Improvement began at once and she made a slow though perfect recovery.

CASE 2. In June, 1881, I was requested by Dr. A. R. Jenkins, to see Annie J—, a prostitute, in consultation with him. She had been suffering from menorrhagia for about three weeks, the hemorrhage at intervals being profuse. Dr. J. had suspected abortion, but this she positively denied. The morning I was called to see her he had made a speculum examination and found a putrid placenta protruding through the os—this he was unable to get away. The girl was pale and exhausted from persistent hemorrhage, which was still going on. I removed the placenta and the bleeding ceased at once. The same treatment as in the preceding case was resorted to, and she also made a perfect recovery.

CASE 3. I was called in the night hurriedly, to see Mrs. W., March 24th, 1883. She was flooding severely, and having regular labor pains at intervals of ten minutes. She was a young primipara and only eight weeks pregnant. The os was dilated to the size of a nickel, and the ovum could be felt protruding. Under the influence of ergot and a tampon, the pains increased in rapidity and strength and the fœtus was soon expelled. It was about the size of my finger. I waited for the expulsion of the membranes; gave more ergot; the pains were excessively severe; she suffered from them much more than during the delivery of the fœtus. I finally tamponed the vagina and left for home, hoping they might be expelled during the night. The next morning I removed the tampon and could distinctly feel the membranes high up in the uterus. She was feeling very well, and wanted to get up. I gave her more ergot, washed out the vagina with carbolized water, and put in a fresh tampon. The pains excited by the ergot were so distressing that the husband called upon me three times during the morning, urgently requesting that something be done to quiet his wife's sufferings. In the evening I combined McMunn's Elixir of opium with the ergot, and gave forty drops of each. The pains became so unbearable that I finally had to administer morphia hypodermically, to quiet her agony. The next morning great sensitiveness had developed over the uterine region. She lay with her knees drawn up, had dry, brown tongue, complete loss of appetite and chilly sensations up and down the spine. The pulse and temperature were not above 80. No more hemorrhage occurred; there was the faintest odor upon the finger after examination. The os, under the contractions produced, had become so small that I could scarcely get the tip of my finger through. I gave no more ergot. That afternoon Dr. Jenkins, at my request, administered chloroform, and I dilated the os and removed the partially decomposed membranes. Antiseptic vaginal injections were used several times daily as long as any odor or discharge remained, and nothing more was done except to give tonics and nourishment.

My attention was especially called to the unusual effects of the ergot upon the circular fibres of the internal os, in this case. The pains produced by it are simply terrible. The patient was only eight weeks pregnant. The os, which had previously been sufficiently patulous to allow the admission of the index finger, with which the mass of membranes could be distinctly felt, was under the influence of ergot almost completely closed, and remained so as long as it was given. The very means used for exciting more powerful uterine contractions seemed to be the cause of the failure to effect their expulsion.

The circular fibres near and about the internal os appeared to receive the effect of the oxytocic first, and when the fundus and body of the womb contracted, the advance of the contained membranes was resisted, and the pains were intensified and rendered of no effect. This lady made a narrow escape. The error was in not persisting in the removal of the secundines immediately after the delivery of the fœtus. There probably would have been some difficulty in their complete removal, but the pain and the risk would have been *slight* in comparison to the agony which she suffered from the effects of the ergot, and the risks of impending anæmia and blood poisoning.

I can readily understand how we might feel justified in permitting delay in some cases, when there is great nervous excitement and exhaustion. These might, perhaps, better be allayed or overcome by appropriate treatment, keeping a careful watch in the meantime for symptoms indicative of trouble, and acting promptly should they arise. Hemorrhage might have been so excessive as to make delay for a short time the wiser plan. The added shock of immediate removal might better be delayed until reaction set in.

The point which I wish to emphasize is, that the patient is not *safe* until her uterus is empty and firmly contracted, and that it is the duty of the attending physician to see that these ends are accomplished before he leaves the case.

The President of the New York Obstetrical Society,—Dr. Skene—in 1878, when this subject was being discussed, said : "The sooner the uterus is emptied the sooner can the physician feel that the patient is safe. If the cervix is not dilated, which is a rather rare occurrence, and there is no hemorrhage, it should be dilated at once, and the placenta removed." If left behind, and serious trouble ensues, the responsibility should be placed upon the physician.

A septicæmia once set up by the absorption of putrid material cannot always be relieved by its speedy removal and the vigorous use of antiseptics. It may be too late. The general principle, then, of securing the complete and speedy removal of the contents of the uterus and its firm contraction after abortion, I insist upon as the only safe practice. The rare exceptions demand the closest watchfulness.

---

**Dr. A. Tepliashin** (Vratch, 1882, No. 35), recommends a thin stream of cold water from a teapot lifted from one to one and a half feet from the abdomen, in cases of colic. He has seen it relieve pain when opium and morphia had failed.—*Ex.*

